# Lexical development of noun and predicate comprehension and production in isiZulu

**DOI:** 10.4102/sajcd.v63i2.169

**Published:** 2016-07-28

**Authors:** Ramona Kunene Nicolas, Saaliha Ahmed

**Affiliations:** 1 Department of Linguistics, University of the Witwatersrand, South Africa

## Abstract

This study seeks to investigate the development of noun and predicate comprehension and production in isiZulu-speaking children between the ages of 25 and 36 months. It compares lexical comprehension and production in isiZulu, using an Italian developed and validated vocabulary assessment tool: The Picture Naming Game (PiNG) developed by Bello, Giannantoni, Pettenati, Stefanini and Caselli (2012). The PiNG tool includes four subtests, one each for subnoun comprehension (NC), noun production (NP), predicate comprehension (PC), and predicate production (PP). Children are shown these lexical items and then asked to show comprehension and produce certain lexical items. After adaptation into the South African context, the adapted version of PiNG was used to directly assess the lexical development of isiZulu with the three main objectives to (1) test the efficiency of the adaptation of a vocabulary tool to measure isiZulu comprehension and production development, (2) test previous findings done in many cross-linguistic comparisons that have found that both comprehension and production performance increase with age for a lesser-studied language, and (3) present our findings around the comprehension and production of the linguistic categories of nouns and predicates. An analysis of the results reported in this study show an age effect throughout the entire sample. Across all the age groups, the comprehension of the noun and predicate subtests was better performed than the production of noun and predicate subtests. With regard to lexical items, the responses of children showed an influence of various factors, including the late acquisition of items, possible problems with stimuli presented to them, and the possible input received by the children from their home environment.

## Introduction

Research on the emergence of early lexical comprehension and production is very important for both enhancing our understanding of language acquisition and diagnostic purposes. In the South African context, improving numeracy and literacy skills remains a huge challenge in the public education sector (Department of Basic Education, 2014). Language policies favour L1 learning in the foundation years (ages 6–9 years), yet psycholinguistic measures of children’s competencies are not included in either educational or political initiatives. Existing literature is very limited regarding the cognitive performance of children who speak a South African indigenous language (also linguistically classified as a Bantu language) in their early language acquisition.

The other challenge is finding locally appropriate standardised tools that are relatively easy to use in any given language. It is heartening, however, to note that several researchers in speech and language therapy have adapted several international standardised tests to the local context and are starting to include crucial information in the measurement of children’s typical and atypical development. Having normative data allows for interventions in delayed or impaired language as well as effective language teaching and learning (Kathard *et al*., 2011; Pascoe & Smouse, 2012).

Despite an increase, there are still too few academic studies on the comprehension and production of South African Bantu languages. Those available include studies focusing on phonological development (Gxilishe, 2004; Pascoe & Smouse, 2012); nominal morphology (Gxilishe, 2008); morphosyntactic development of the noun classes (Kunene, 1979; Tsonope, 1987); the development of the passive construction (Bortz, 2013; Demuth, 2003); and pragmatic development in late language acquisition (Kunene-Nicolas, 2015).

Although these studies have greatly advanced our knowledge of less-studied languages, no one has managed to present comprehensive linguistic research on the developmental aspects of the lexicon inventory of the Bantu-speaking child, similar to that regarding children who speak western languages, such as English, French, and Italian. Researchers tend to focus on particular aspects within linguistic theory (Berko Gleason & Bernstein Ratner, 1993, pp. 326–327). To date, the available literature on early language acquisition has confirmed, through the study of many languages from different language typologies, that certain stages of language acquisition are universal (Harley, 2014). It is, therefore, widely accepted that all children begin their trajectory towards language through comprehension, while their motoric and cognitive apparatus lags in terms of language production.

This study seeks to investigate the development of noun and predicate comprehension (PC) and production in isiZulu-speaking children between the ages of 25 and 36 months. The study is part of an international research collaboration that aims to investigate speech and co-speech gesture production and comprehension development in children. It compares lexical comprehension and production of two romance languages, Italian and French, and two South African Bantu languages, isiZulu and Sesotho, using an Italian developed and validated vocabulary assessment tool. This paper presents the preliminary findings of the lexical development of isiZulu speakers.

## The lexicon: Nouns and predicates

We use words to communicate about everything related to our physical environment, including events, activities, people, objects, places, relations, properties, and states of being (Clark, 1995, p. 1). Words stored by language users are drawn from the lexicon that can also be understood as our mental dictionary. Bates and Goodman (1997) found that, as children transition from the first word stage to sentences and extended discourse, while learning productive control over the basic morphosyntactic structures of their native language, the emergence and elaboration of grammar are highly dependent upon vocabulary size. The lexicon is therefore linked to phonology, comprehension and production, and grammar (Gentner, 1982). The child’s lexicon is dependent on the development of meaning construction and categorisation skills (Markman, 1991). Measuring comprehension and production of nouns and predicates is increasingly used as an important diagnostic and prognostic tool in atypical populations (Stefanini, Bello, Caselli, Iverson & Volterra, 2009). As such, the MacArthur Communicative Development Inventory (CDI) was initially developed to study the relationship between the lexical and grammatical development of English-speaking children (Bates, Dale & Thal, 1995; Fenson *et al*., 1994; Fenson, Marchman, Thal, Dale & Reznick, 2007) but has since been adapted for use in more than 62 languages.

It is well documented that typically developing children are accurately able, by 3.5 years of age, to produce most of the basic morphosyntactic structures of their languages such as relative clauses, the passive construction and other complex forms (Bates & Goodman, 1997; Demuth, 2003). Our present study seeks to look at one component of the lexicon: the comprehension and production of the nouns and predicate categories of words in isiZulu. Nouns and predicates are characterised by differences in their perceptual and cognitive complexity (Davidoff & Masterson, 1996; Gentner, 1982; Gentner & Boroditsky, 2001), which leads to distinct mental representations (Slobin, 2008).

In the cross-linguistic study by Caselli *et al*. (1995) the authors highlight the noun-verb sequence in early acquisition as proposed by the ‘Whole Object Constraint’ (Markman, 1991). Gentner (1982) reported the late appearance of verbs, which are more complex in structure than the underlying semantic structure of nouns. O’Grady (1987) pointed out that nouns are used as ‘arguments’ or ‘primaries’ that refer to entities or a class of entities, whereas verbs and adjectives are often used as predicates or ‘secondaries’ (Caselli *et al*., 1995, p. 162). This means that, for a child to produce verbs and adjectives successfully, nominal arguments have to be in place. The acquisition of verbs will therefore be affected by the child’s mastery of nouns. These theoretical arguments, however, have been challenged recently by the appearance of language groups that show children mastering verbs at a faster rate in Mandarin (Cheng, 1994) and Korean (Gopnik & Choi, 1995). Literature does, though, confirm that verbs, adjectives, and function words appear later in early child acquisition (Caselli *et al*., 1995). This study therefore seeks to document the development of the noun and predicate in isiZulu.

## Bantu language acquisition

Bantu languages are typologically similar and share several typical grammatical features. IsiZulu is a South Eastern Bantu language of the Nguni cluster spoken primarily in South Africa (especially the southeastern areas of Kwa-Zulu Natal), but it also has speakers in neighbouring countries. IsiZulu is highly mutually intelligible with other Nguni languages, such as isiNdebele, isiXhosa, and siSwati. In 2011, South Africans citing isiZulu as their home language numbered 11.5 million, or 22.7% of the population, the language that has the highest number of speakers (Census, 2011).

IsiZulu is a Subject-Verb-Object (SVO) language with a high number (about 15) of noun classes, triggering the agreement of verbs, adjectives, and other elements. In other words, ‘nominal and verbal modifiers follow the noun and verb respectively, and grammatical morphology is prefixed to both nouns and verbs’ (Demuth & Suzman, 1997, p. 2). The subject can be dropped and it is therefore a pro-drop language as well (Gxilishe, Villiers & Villiers, 2007; Kunene, 2010; Suzman, 1985, 1991). It has a very rich system of tense and aspect. These are expressed in a variety of simple tenses with optional aspectual affixes, compound tenses allowing composition of many of the simple tenses, and a large number of auxiliary verbs (Buell, 2005, p. 6).

Demuth (2003, pp. 1–4) gives a thorough overview of South African Bantu language acquisition studies of siSwati, isiZulu, isiXhosa, Setswana, and Sesotho. Most studies have looked at the noun class prefix and nominal agreement, consonants and clicks, acquisition of word order, relative clauses, and morpho-phonology (for a review on studies of Bantu language acquisition, see Demuth, 2003; and for a contemporary overview of studies on SA Bantu language see Gxilishe, 2008; Pascoe & Smouse, 2012). From existing literature, we know that child speakers of isiZulu, Sesotho, siSwati, and isiXhosa have fully acquired the nominal class system by the age of three years. We also know, from a study of isiXhosa-speaking children (Gxilishe *et al*., 2007), that the plural agreement is better produced than the singular subject agreement.

Despite the numerous studies on the isiZulu verb (or related languages), we have not come across literature that documents the acquisition of verbs, adjectives, and adverbs or the noun and its morphology.

Studies on South African Bantu languages are definitely increasing, but as yet there has been no study that has looked at simultaneous comprehension and production during lexical development.

## Aims and objectives

We focus on the lexical development of comprehension and the production of nouns and predicates from a speech perspective. Specifically, this article seeks to explore the lexical development of isiZulu using the adapted assessment tool with three main objectives. These are to:

- Test the effectiveness of the adaptation of a vocabulary tool to measure isiZulu comprehension and production development.
- Test the universal finding that both comprehension and production performance increase with age for a less-studied language, isiZulu.
- Present our findings on the comprehension and production of the linguistic categories of nouns and predicates.

## PiNG assessment tool

Early childhood development research shows a strong interdependence between vocabulary, phonology, and grammar in both typical and atypical populations (Marchman & Thal, 2005; Stoel-Gammon, 2011). If children who show delays in their expressive vocabulary repertoires can be identified in time, this could assist in early intervention for children at high risk for language impairment, as shown in the studies by Desmarais, Sylvestre, Meyer, Bairati and Rouleau (2008) Ellis and Thal (2008).

Constructed and validated in Italy, the Picture Naming Game (PiNG) was specifically developed to assess lexicon production and comprehension in children between the ages of 19 and 37 months, involving the consideration of both nouns and predicates, and based on the Italian MB-CDI. Previous studies have shown that it is extremely relevant to investigate the relationship between vocabulary comprehension and production as well as between nouns and predicates, as these skills and their relationships are indicators of both the level of language development and conceptual organisation. In general, studies using the PiNG tool with Italian children proved that the comprehension subtests were easier than the production subtests, thus allowing for their administration in younger children and resulting in fewer errors. Similarly, children found the noun subtests easier than the predicates subtests. Therefore, lower variability was found in vocabulary comprehension compared with production, and in nouns compared with predicates for hearing children (Bello *et al*., 2012; Rinaldi, Caselli, Di Renzo, Gulli & Volterra, 2014).

The PiNG tool consists of two sets of colour pictures and contains two tasks; comprehension and production tasks which in turn contain four subtests. The first set has 22 images (20 test pictures and two pre-test pictures) of objects and tools, animals, food and clothing (e.g. a fork, a lion, bananas, gloves) and is used in evaluating the comprehension and production of nouns in the noun comprehension subtest (NC) and the noun production subtest (NP), respectively. The second set contains 22 images (20 test pictures and two pre-test pictures) showing actions, location adverbs, and/or adjectives (for example, to push, close by or far away, big or small) and is used to evaluate the comprehension and production of predicates in the PC subtest and the predicate production subtest (PP), respectively. The original PiNG test for Italian children was adapted from the Italian MB- CDI and the items had different levels of difficulty. It included items that were ‘easy’, ‘moderately easy’, and ‘difficult’, based on the Italian normative sample (Bello *et al*., 2012; Pettenati, Sekine, Congestri & Volterra, 2012; Pettenati, Stefanini & Volterra, 2009; Stefanini *et al*., 2009; Stefanini, Recchia & Caselli, 2008).

In this paper, we report on the adaptation of PiNG to isiZulu. The PiNG tool has already been successfully adapted to other languages and cultures. For example, a study by Pettenati and colleagues provided the first occasion for a cross-cultural comparison of gestures and vocabulary production and comprehension of 22 Italian and 22 Japanese children between 25 and 37 months of age (Pettenati *et al*., 2012). The PiNG tool was also used to assess vocabulary production and comprehension in toddlers in a study carried out in Australia by Hall, Rumney, Holler and Kidd (2013). The Australian study focused on a group of 50 typically developing children between 18 and 31 months of age, investigating the interrelationship between play, gesture use, and spoken language development.

## Methods

### Stage 1: Translation of the PiNG lexicon into the target languages

Translation of the set of nouns (20 target nouns in the comprehension task +20 target nouns in the production task +2 × 2 = 4 lexical items for the pre-tests) and the set of predicates (20 target verbs and/or adverbs and/or adjectives in the comprehension task +20 target verbs and/or adverbs and/or adjectives in the production task +2 × 2 = 4 lexical items for the pre-tests) was carried out in isiZulu by the researcher, who is a native speaker of isiZulu and a linguist, together with two isiZulu-speaking research assistants, who are also linguists. The translation was further tested in a pilot study of native Zulu adults for validation (see Stage 2).

Of particular interest in the international collaborative comparative study is the different language typology of romance languages that are analytic and Bantu languages that are agglutinative. In the initial adaptation, a conscious decision was made to adapt the protocol questions as closely as possible to the original Italian version, that is, questions were to be ‘neutral’ so as not to give a clue to the answer. For example, in the Italian version, the question would be translated to ‘show me running’, which does not provide any clue to the participant for the comprehension task and, therefore, the speaker cannot get a clue on the referent. A participant could choose any item he or she deemed fit. However, because of the Noun Class system, agreement concords, and morphosyntax structure of Bantu languages, the question must have the relevant noun class and subject concord, which may give a clue to the item. For instance, in isiZulu, a semantic translation for the above example would read *ngikhombise ogijimayo* (show me the person that is running): this would not allow a speaker to select a different card in the task because the question requires the speaker to select a card with a person who is doing something, for example, running.

### Stage 2: Pilot study with adults

Twenty two adults (11 males and 11 females) participated in the Zulu adult pilot study. Participants were drawn from the pre-dominant isiZulu speakers of Kwa-Zulu Natal, the south-east region of South Africa. Participants were university students from various communities in the Kwa-Zulu Natal area, 60% of whom were from the Empangeni area. The other 40% were from surrounding areas: Pongola, Harrismith, Durban, Eshowe, and Pietermaritzburg. Applying a neutral questioning style did not work. Participants would reformulate the question or stop the interviewer to ask for more clarity. If the questions were amended, participants answered with no difficulty. The inclusion of the class prefixes did not affect the results but rather assisted the participant in understanding what was requested of him or her. This was indicated by the fact that once the correct class prefixes were used, the participants would indicate that they did not recognise an item, or they would give an answer if they did. With the neutral questions, the participant would simply halt the interview, seek clarification, and personally supply the class prefixes. When the interviewer asked why the participant reformulated the utterances, all participants stated that the ‘neutral’ utterances were grammatically correct, but ambiguous and confusing. It is interesting to note that all 22 participants corrected the utterances.

Agreement between participants was 95% for the comprehension subset and 86% for the production subset. Four items under the NP subset produced either no responses or ‘I do not know’ answers, referring specifically to bidet, radiator, penguin, and seal. Under the PP, two predicate task words produced a low frequency of correct target words (spinning, heavy, far apart).

### Stage 3: Modification of the material

From the adult pilot results, it became clear that the isiZulu version of PiNG needed further adaptation before the pilot study with children was initiated. The adults seldom produced words in isiZulu for ‘seal’ and ‘penguin’ and so these items were changed to ‘snail’ and ‘crocodile’, respectively. As both ‘radiator’ and ‘bidet’ are foreign cultural objects, these two items were replaced by ‘heater’ and ‘toilet’. Some pictures were specifically cultural, such as the picture of the ‘roof’, which was a European type of roof, but in order to allow a systematic comparison with other languages in the four-language project, some items were retained for future adaptation.

### Stage 4: Pilot study with children

After the changes to the above-mentioned picture items, a pilot study was conducted with 15 Zulu children. The group included five children aged 25 months (±1 month), five children aged 30 months (±1 month), and five children aged 36 months (±1 month). This was done in order to test the corresponding adaptation of PiNG on a small sample in case further adaptation was needed before going onto the main study. Participants were drawn from Kwa-Zulu Natal, the same area where the adult pilot study was conducted. Data was collected from the urban townships of Empangeni and Ngwelezane on the northern coast of Kwa-Zulu Natal. The principals and caregivers were very helpful in providing the researchers with the children’s clinic cards. These vaccination cards aided the researchers in selecting participants for the appropriate age cohorts. The files provided by the teachers also gave the researchers additional information, including, for example, whether a child had been born prematurely or had a learning disability and needed to be excluded from the selected participants.

We worked with nine crèches to ensure that our three age groups were exact. Many crèches could not form part of our participant sample because those children were bilingual and would alternate between naming items in English and isiZulu. We finally chose two schools from the city centre of Empangeni and four schools in the Ngwelezane township. Administration of the PiNG tool began with a familiarisation phase that involved playing various card games, counting games, and naming games with the children. Researchers also played with the children in the school playgrounds on the swings and slides. Once the researchers felt that the children were comfortable enough, they asked the children to play a game with them in front of the camera.

## Main study

The main study for the isiZulu data was collected from Soweto in the Gauteng province. The move from KZN was purely logistical as the researchers were all Gauteng based. Monolingual isiZulu-speaking children were chosen with the help of their caregivers. Children’s vaccination cards were examined to exclude premature babies or those with any recorded pathologies. All crèches require clinic or vaccination cards in order to enrol the child. The caregivers also assisted in selecting children, who they said showed no language delays in comparison to their peers. All selected children had parental consent (see the Ethics section).

Forty-nine children from four neighbouring crèches participated in the study. Nine children were excluded from the data sample for various reasons: some children did not complete the two tasks, some children were bilingual and code-switched regularly, some children spoke too softly for the camcorder to record sound, and one child was sleepy and had to go for a nap. For this study, 36 participants were chosen with 12 per age cohort in consideration of gender balance. There were a total of 19 females and 17 males across the different age cohorts (Table 1).

**TABLE 1:** Zulu participants age groups.

Group in months	Age range		Average age m; d	SD m; d	Number of children	
	
	Min	Max			Female	Male
25	23.08	26.14	24; 29	0m; 29d	7	5
30	28.01	31.26	30; 4	1m; 13d	7	5
36	34.16	38.29	36; 13	1m; 7d	5	7

### Procedure

The procedure of this study followed those of previous studies (Bello *et al*., 2012; Pettenati *et al*., 2009, 2012; Stefanini *et al*., 2008, 2009). The tool began with the comprehension task picture, followed immediately by the production-eliciting picture of the noun sets. There was a short break after the noun items and then the predicate items would be elicited, again starting with the comprehension task picture, followed by the production-eliciting picture of that set. The following section details the procedure that was followed in our study.

After the familiarisation period, during which we played different card games with the children, all children were tested individually at their schools. Three sets of pictures per set were presented to each child on a small table. The first part of the task was comprehension, in which a child was asked ‘Where is the cat? Show me the cat’ for a noun comprehension item or ‘What is this child doing? What is this one doing?’ for the PC item. The second part of the each subset was production, in which the child was asked, ‘What is this?’ for the NP subset or ‘What is he doing?’ for the PP subset. The third card was a distractor to eliminate the choice by luck. A total of 22 cards were presented for the noun subtest, and another 22 cards were presented for the predicate subtest. The first two sets were pre-test cards to ensure that the child understood what was expected. The data was based on the remaining 20-card set per subtest. For the comprehension task, only one prompt was considered. For the production task, if the child struggled with producing the correct item, a second prompt was used. All elicitations were filmed for later data coding and analysis.

Two research assistants and the researcher of this study collected the data. All researchers are first-language, native speakers of isiZulu, linguists with fieldwork experience in the collection of data from children.

### Coding and transcription of the data

The coding system was adapted from previous studies (Bello *et al*., 2012 Pettenati *et al*., 2012). All the children’s responses were coded later from the video data with an annotation system that was designed for the purpose of coding for gestures as well as for wordings from the child and the experimenter. All tasks administered to the children were coded on *ELAN,* a linguistic annotation tool created by the Max Planck institute (*ELAN,* n.d.; Wittenburg, Brugman, Russel, Klassmann & Sloetjies, 2006).

For the comprehension subtests (NC and PC), if the child indicated (either by pointing, showing, or verbalising) the photograph corresponding to the item indicated by the adult researcher, the answer was considered correct. If the child selected the no target photograph or did not respond at all, the response was coded as incorrect or no response, respectively. Similarly, in the production subtests, if the child produced the target lexical item, their response was coded as correct. If the child produced a non-target item or did not respond at all, their response was coded as incorrect or no response, respectively. For some photographs, more than one answer was accepted as correct; for instance, for the ‘diaper’ item, some children called it *ipampers* referring to the brand of disposable nappies or ‘ikhimbi’ also referring to a brand of disposable nappies. For the production subtest, children were prompted twice if their initial response was incorrect. If a correct response was produced after the second prompt, the answer was considered to be correct.

Synonymous items were considered to be correct synonyms, for instance *ilorri* (a lorry) and *itruck* (a truck) for the truck item. Responses that had a semantic relationship to the item depicted were coded as NTS, no target, but semantically correct to measure if the concept was in place even though the production was not successful. Incorrect responses occurred where the production was not the target response nor semantically related, for instance, *isidudu* (motorbike) for ‘truck’.

### Validation and reliability

Three trained native isiZulu-speaking research assistants and the researcher independently coded the verbal transcription, that is, orthographical transcription directly from the film footage. Two different research assistants, who are also trained linguists, coded the classification of the speech responses as well as those of gesture. Disagreements were resolved through discussion.

After the annotation phase was completed, all data was exported to Excel for an ultimate verification (internal consistency on the coding) and statistical analysis.

## Ethical considerations

Ethical considerations guided the pilot study as well as the main study. All children who participated were recruited on a voluntary basis after their caregivers signed an informed consent form, which was provided in their language, and after they themselves agreed to participate at the start of the task (‘Nouns’ or ‘Predicates’). Parents or members of the crèche were welcomed in the room during the administration of the tool. The tasks were interrupted or ended if a child verbalised a desire to stop, or expressed discomfort by crying and/or withdrawing. Children’s identities were kept confidential, and data obtained from this project were not disclosed to any third party. The Wits HREC Non-Medical Ethics Committee granted ethical clearance for the study (protocol number H13/08/43).

## Results

Children’s responses were analysed according to the coding criteria listed in the method section. For our first objective, we analysed the comprehension and production tasks across the three age groups to test whether the PiNG assessment tool was effective in detecting the development of comprehension and production in isiZulu. Our second objective overlapped our first, and so our first finding addresses both of our objectives.

For the comprehension and production tasks, an analysis of variance, ANOVA, was run with the age group as the independent variable.

The correct answers for the comprehension and production task items are illustrated in Table 2.

**TABLE 2:** Comprehension and production task scores per age group.

Variables	Comprehension task			Production task		
Age group	G25	G30	G36	G25	G30	G36
Mean	12.8	13.6	14.7	8.1	8.8	10.7
SD	2.9	2.4	2.8	2.7	2.5	2.5

A significant difference across the age groups emerged at (*F*[2.69] = 3.143, *p* < 0.05 for the comprehension task. Post-hoc Bonferroni analysis showed that the effect of age was significant at 0.05 among all age groups throughout the entire sample, but was not significant between the 25-month-old and the 30-month-old groups. Similarly, for the production task, there was a significant age effect across the whole sample at (*F*[2.69] = 6.567, *p* < 0.05. Post-hoc Bonferroni analysis showed that the difference was not significant between the 25-month-old and the 30-month-old groups. However the 36-month group performed significantly better than the two younger groups at *p* < 0.003 to the 25-month group and *p* < 0.035 to the 30-month group.

### Lexical item composition

In order to test the comprehension and production tasks per lexical categories, we looked at the performance of the nouns and predicates across the age groups. We performed an ANOVA between groups per lexical subset.

The comprehension of the noun and predicate subtests was better performed than the production of noun and predicate subtests across the age groups. In Table 3 and Table 4, Zulu children performed better at labelling the correct items for nouns (*F*[2,33] = 3.70, *p* < 0.04) than labelling the correct items for predicates (*F*[2,33] = 0.94, *p* < 0.40) with post-hoc test Bonferroni confirming that there was a significant difference between the 36-month group and the 25-month group at *p* < 0.03. There was no significant difference between the 25-month and the 30-month groups, nor between the 30-month and the 36-month groups for the noun subtest. For the PC subtest, post-hoc Bonferroni test confirmed that age had no significant effect, although there was a developmental trend between the different age groups.

**TABLE 3:** Lexical comprehension noun and predicate subtest means (SD) per age group.

Age group	Noun comprehension mean		Predicate comprehension mean	
	
	*n*	SD	*n*	SD
Group 25-months	13.75	2.2	11.75	3.2
Group 30-months	14.67	1.6	12.58	2.8
Group 36-months	16.08	2.5	13.33	2.4

**TABLE 4:** Lexical production noun and predicate subtest means (SD) per age group.

Age group	Noun production mean		Predicate production mean	
	
	*n*	SD	*n*	SD
Group 25-months	7.75	2.3	8.50	3.0
Group 30-months	8.50	1.9	9.08	2.9
Group 36-months	11.42	2.9	10.00	1.8

For the production of nouns and predicates, we noted a similar pattern in that children performed better at labelling the correct items in the NP subtest (*F*[2,33] = 7.4, *p* < 0.002) than in the PP subtest (*F*(2,33) = 0.96, *p* < 0.393). Post-hoc Bonferroni tests confirmed that there was a significant difference between the 36-month group with both the 25-month group and 30-month group, at *p* < 0.003 and *p* < 0.02, respectively. For the PP subtest, the post-hoc Bonferroni test confirmed that age had no significant effect across the three age groups. The 36-month children had an equal chance of correctly labelling PP items as the children in the 25-month and 30-month groups, despite the developmental trend we observe in Table 3 and Table 4.

### Percentage of correct responses per item

For our third objective, we present our findings on the performance of the lexical items. In order to have a better understanding of the performance on the comprehension and production task, as well as noun and predicate subtests, we ranked the items in terms of correctness according to the total sample shown in Table 5.

**TABLE 5:** Percentage of correct answers for comprehension and production provided by (*N* = 36) children.

Variables	Percentage of correct answers by children
**Noun comprehension subtest:**	
Doll	100
Hat	100
Boots	100
Bidet/toilet	97
Motorcycle	94
Spoon	94
Apples	92
Sofa	92
Iron	86
Cow	86
Watch	83
Glasses	81
Box	72
Clouds	53
Terrace	50
Mountain	44
Penguin/snail	44
Elephant	44
Bib	42
Hammer	39
**Noun production subtest:**	
Comb	92
Socks	92
Bananas	89
Bag	81
Umbrella	78
Hen	75
Fork	78
Glass	78
Table	72
Radiator	53
Gloves	33
Diaper	31
Picture	28
Truck	19
Book	19
Beach	14
Lion	8
Seal/crocodile	3
Roof	0
Flags	0
**Predicate comprehension subtest:**	
To pull	94
To sweep	92
To comb	92
To drink	89
To bite	86
Dirty	83
To swing	81
To run	81
To scramble up	75
Big	69
To build	69
High	56
To greet	56
To walk	47
Behind	44
Close	39
Full	31
Outside	31
To embrace	31
Short	25
**Predicate production subtest:**	
To push	94
To drive	89
To eat	89
To phone	83
To wash	72
To play	69
To kiss	67
To open	64
To fall	64
To laugh	61
To swim	53
Small	50
Clean	28
To turn	17
Empty	6
In front of	6
Heavy	3
Far	3
Inside	0
Long	0

#### Comprehension task

Under the noun comprehension subtest, three items were perceived correctly by all 36 children; ‘doll’, ‘hat’, and ‘boots’ had a 100% response rate across all ages. Five items had less than 50% success; ‘mountain’, ‘snail’, ‘elephant’, ‘bib’, and ‘hammer’, meaning that fewer than 18 children across the three age groups found these items ‘difficult’.

The photograph of the mountain showed a rising mountain of the European Alps, a type of geographical feature that is not commonly seen in South Africa. It was interesting to note that the children had difficulty identifying ‘snail’ and ‘elephant’, but managed easily to identify the domestic animals such as ‘cow’. The ‘bib’ and ‘hammer’ were also not easily identified: the children gave answers that focused more on the baby who was wearing the ‘bib’ and did not respond at all to the ‘hammer’ or said, *angazi* (I do not know).

Under the PC subset, there were seven items that had less than 50% success; ‘walk’, ‘behind’, ‘close’, ‘full’, ‘outside’, ‘to embrace’, and ‘short’.

Some of the responses for ‘walk’ were *uhamba kuphi* (walking where?), as the picture showed a young boy walking along the passage of a house. The smaller children would either focus on the clothes the child was wearing or on the distractor card, which showed a young boy playing with toys. This performance did not show a developmental trend, which meant the 36-month-old children also had a similar chance of not perceiving or identifying ‘the walking’ from this picture. The item ‘embrace’ also produced some interesting comments across the age groups, similar to ‘kiss’ found in the PP subtest. The older the children, the more they avoided discussing intimacy with responses like *bayaganga* (they are being naughty) or they would simply avoid looking at the picture and focus on other pictures. The adjectives ‘short’, ‘full’, ‘outside’, and ‘behind’ were very difficult items that showed a distinct developmental pattern with responses that were semantically related, so for ‘short’, a child would say *yincane* (it is small) referring to a short pencil in the picture, and yet the prompt was for them to point to the object, which does not necessitate a verbal response.

#### Production task

Overall, production items scored lower than comprehension items, as seen in Table 5. Under the production subtests, for the NP, the items ‘comb’ and ‘socks’ had the highest response rate at 92%, with children from all age groups correctly labelling these items. Ten items had a success rate of less than 50%; the items were: ‘gloves’, ‘diaper’, ‘picture’, ‘truck’, ‘book’, ‘beach’, ‘lion’, ‘crocodile’, ‘roof’, and ‘flags’.

The 36-month-old group was more familiar with the leather gloves depicted in the picture with most children saying *izandla/into yezandla zikamama* (hands or something for my mum’s hands). Glove sizes in South African shops start for children who are about 4 to 5 years of age, and mittens are not commonly found. The older children labelled the item ‘diaper’ correctly, compared with the young children who tended to call it *ipenti* (a panty).

The ‘picture’ item was also more familiar to the 36-month-old group, even though most children across the age groups identified it as a TV, because the picture looked like a flat screen TV. The picture depicted a lone beach with a blue sky, the ocean, and a strip of sand. The picture also garnered ambiguous responses from the adults in the pilot study.

The item ‘truck’ produced the semantically related ‘a car or a bus or a big car’. This item did not display any developmental trajectory, as 36-month-old children were equally likely to produce ‘car’ rather than the target word *itruck* or *ilori*.

The ‘book’ item mostly produced *ibhayibheli* (a bible) across age groups as well. The ‘beach’ item produced equally random responses, with children either focusing on the water in the photo or on the sky.

The wild animals ‘lion’ and ‘crocodile’ produced very interesting responses such as *ikgokgo* (a monster) or some onomatopoeia sounds illustrating that ‘this is a scary thing that will eat me’. A few children avoided even looking at the picture or quickly pushed the card to the researcher.

The ‘roof’ item depicted a European type roof and a portion of a house with trees surrounding it. Most responses were either *indlu* (house) or *isihlahla* (a tree). This picture did not reveal an age pattern because the responses were random. The item ‘flags’ was extremely difficult, with no child giving a correct response. Most responses were *iAfrica* (Africa) or *yiduku* (head scarf), and these semantically related responses increased with age.

In the PP subtest, the following seven items were difficult for the children: ‘to turn’, ‘empty’, ‘in front of’, ‘heavy’, ‘far’, ‘inside’, and ‘long’.

The item for ‘to turn’ depicted a group of children on a merry-go-round. Children’s responses included *bayadlala/bahleli* (they are playing or they are sitting). This response did not reveal a developmental trajectory because the responses were random across all age groups.

For the adjectives and adverbs ‘empty’, ‘in front of’, ‘far’, ‘inside’, and ‘long’, the responses did not show correct labelling across all ages. These items showed a developmental pattern when considered in the light of that the responses showed that the children acquired this concept with age.

Interestingly enough, five of these items were in the same set at the PC subtest, which shows consistency in the production of these categories. All the children had difficulty perceiving the item ‘heavy’. This item depicted a young child carrying a brown torn box while grimacing. Children’s responses focused on the child or the box being torn.

## Discussion

Our study begins with an Italian picture naming assessment tool being adapted to a Bantu language, isiZulu. The tool is designed to directly observe the lexical composition of vocabulary in two related tasks; comprehension and production. To our knowledge, most studies on isiZulu or related Bantu languages have either directly observed either comprehension or production, but never both at the same time. Several pilot studies in both adult and children populations enabled us to alter obvious elements of cultural bias. Although some other problematic items remained, we decided to preserve our initial goal by keeping as many items as necessary for our systematic comparison with two romance languages, Italian and French, as well as another South African Bantu language, Sesotho.

Certain items such as the picture of a ‘roof’ did not depict a ‘roof’ as seen by many South African children. Some items such as ‘to turn’, which depicted a merry-go-round produced unexpected results in that one would expect most children to have been exposed to a merry-go-round as these are commonly found in parks, but instead the children focused more on the people in the picture than on what they were doing. Despite the cultural differences that may have stemmed from the images of our stimulus, we note that our findings confirm what has been long documented in literature: that comprehension comes before production. In a related cross-linguistic comparison, Japanese children showed a lower lexical production compared with the Italian children (Pettenati *et al*., 2012) which resulted in the authors finding that cultural factors could influence the design of the test which was originally for Italian children.

In terms of development, our results show how age affects both comprehension and production: 36-month-old children performed better than 30-month-old children who in turn performed better than 25-month-old children. Our statistical analysis did not discern a significant difference between the 25-month-old group and the 30-month-old group for comprehension, but it detected a significant difference between the 36-month to the 25-month and 30-month groups in terms of production. This was not surprising as, in the first study by Bello *et al*. (2012), it was found that noun comprehension increases between 19 and 30 month, followed by a plateau. The Zulu children also showed this plateau, which meant there was little difference between the 25-month and 30-month groups. The PC showed a similar trend, even though the scores were lower than those of the noun comprehension. It is, however, very interesting to note that there was no significant difference in PC among the groups, which may mean that predicates in this task, with the exception of adverbs and adjectives, may be mastered earlier. Gxilishe *et al*. (2007) found that isiXhosa-speaking children between 24 and 30 months correctly employed subject agreement markers on different verb roots.

In terms of production, our findings showed an overall effect of age, which meant that production does indeed lag behind comprehension. Moreover, the larger number of culturally foreign pictures in the noun comprehension subtest suggests that it may be necessary to further adapt the assessment tool to more effectively evaluate the isiZulu lexicon.

Children used a semantic description strategy to try and explain unfamiliar items, which shows that though they may have the concept, the lexical item is not ‘concrete’ enough for them to relate to their physical environment. If perception is difficult, it is harder to retrieve the semantic representation from the lexicon and, as such, retrieval will be impaired (Harley, 2014).

The production of the noun category showed an age effect but the predicate category did not show a similar difference. The higher number of adverbs and adjectives in the PP subtest was difficult for all the age groups, which could explain the lack of a significant difference. Alternatively, if verbs are acquired earlier, it shows that the children have reached a plateau phase between the ages of 24 and 36 months. Further research on the vocabulary spurt in isiZulu would shed more light on this issue.

### Limitations

The lack of a child inventory like the MacArthur Bates CDI for isiZulu and related languages is a disadvantage. We have no idea which items are acquired first, nor do we have the exact age of the vocabulary spurt in isiZulu. Future analysis should look at the gender effect. Although gender-related data is available in this study, it has not yet been analysed. It would be interesting to see whether girls have an advantage over boys, as has frequently been reported in western languages.

### Conclusion

Developing normative lexical data on Zulu-speaking children or those speaking other related Bantu languages is important for research for both acquisition and clinical purposes. Finding a standardised assessment tool that can be used for South African Bantu languages is an ongoing challenge. The PiNG assessment tool has proved to be robust and effective in directly assessing a child’s lexical vocabulary. For further research into isiZulu, the tool would need further adaptation by replacing some images with items of local content.

The literature states that children begin with language comprehension and, when the motoric and cognitive apparatus develops, language production follows suit. This is a universal linguistic parameter. Children start with objects and events around their immediate environment and quickly learn people’s names, concrete objects around them, and familiar routines coming from their home environment. The first words will therefore largely depend on this input, and cultural as well as linguistic constraints may affect this development.

This study shows that as children get older, comprehension and production improve. It would therefore be very important for researchers or speech therapists to factor input into their intervention therapies. Children will understand and talk more about what they know and what surrounds them. Some linguistic phenomena like adjectives and adverbs are complex and not yet acquired by the Zulu child at 36 months.
